# Ceramide Risk Score in the Evaluation of Metabolic Syndrome: An Additional or Substitutive Biochemical Marker in the Clinical Practice?

**DOI:** 10.3390/ijms241512452

**Published:** 2023-08-05

**Authors:** Antonello E. Rigamonti, Michele Dei Cas, Diana Caroli, Adele Bondesan, Silvano G. Cella, Rita Paroni, Alessandro Sartorio

**Affiliations:** 1 Department of Clinical Sciences and Community Health, Università degli Studi di Milano, 20129 Milan, Italy; silvano.cella@unimi.it; 2 Department of Health Sciences, Università degli Studi di Milano, 20142 Milan, Italy; michele.deicas@unimi.it (M.D.C.); rita.paroni@unimi.it (R.P.); 3 Istituto Auxologico Italiano, Istituto di Ricovero e Cura a Carattere Scientifico (IRCCS), Experimental Laboratory for Auxo-Endocrinological Research, 28824 Piancavallo-Verbania, Italy; d.caroli@auxologico.it (D.C.); a.bondesan@auxologico.it (A.B.); sartorio@auxologico.it (A.S.); 4 Istituto Auxologico Italiano, Istituto di Ricovero e Cura a Carattere Scientifico (IRCCS), Experimental Laboratory for Auxo-Endocrinological Research, 20145 Milan, Italy

**Keywords:** metabolic syndrome, obesity, ceranides, CERT1, cardiovascular disease risk

## Abstract

Ceramide risk score (CERT1, ceramide test 1), based on specific ceramides (Cers) and their corresponding ratios in the plasma, has been reported as a promising biochemical marker for primary and secondary prediction of cardiovascular disease (CVD) risk in different populations of patients. Thus far, limited attention has been paid to metabolic syndrome, a condition considered at high CVD risk. The aim of the present study was to evaluate CERT1 in a group of obese subjects without (OB-MetS−) and with (OB-MetS+) metabolic syndrome (according to the International Diabetes Federation (IDF) diagnostic criteria), compared to an age- and sex-matched normal-weight (NW) group. In all participants, plasma levels of Cer 16:0, Cer 18:0, Cer 24:1, and Cer 24:0 were measured, and the corresponding ratios Cer 16:0/24:0, Cer 18:0/24:0, and Cer 24:1/24:0 were calculated together with CERT1. Subjects with obesity showed higher CERT1 values than the NW group (*p* < 0.05), with no difference between OB-MetS− and OB-MetS+ groups. Waist circumference (WC), homeostatic model assessment of insulin-resistance (HOMA-IR) (surrogates of IDF diagnostic criteria for metabolic syndrome), and C reactive protein (CRP) (a marker of inflammation) were predictors of CERT1 (*p* < 0.05), with the contribution of the other IDF criteria such as arterial hypertension and dyslipidemia being negligible. Adjustment for WC resulted in a loss of the difference in CERT1 between OB-MetS− and NW subjects, with the combination of WC and HOMA-IR or CRP as covariates being necessary to yield the same effect for the difference in CERT1 between OB-MetS+ and NW subjects. Importantly, an association was found between CERT1 and vascular age (VA) (*p* < 0.05). Proportions of NW, OB-MetS− and OB-MetS+ subjects appeared to be distributed according to the CERT1-based risk groups (i.e., low, moderate, increased, and high risk; *p* < 0.05), with some OB-MetS− subjects included in the increased/high-risk group and some OB-MetS+ in the low/moderate-risk one. In conclusion, the clinical diagnosis of metabolic syndrome seems to be inaccurate to assess CVD risk in the obese population; however, further studies are needed before considering CERT1 as an additional or substitutive biochemical marker in clinical practice.

## 1. Introduction

Ceramides are bioactive lipids belonging to the sphingolipid family that, interacting with receptors, enzymes, and intracellular signaling proteins, exert a pivotal role in cellular physiology/pathophysiology such as glucose homeostasis, lipid metabolism, endothelium function, adipogenesis, cell apoptosis, and inflammation [1,2,3,4,5].

During the past decade, ceramides have been demonstrated to be not only of great interest for biochemical and pharmacological research due to the implications in obesity and related comorbidities, but also of diagnostic utility in clinical practice [6].

In particular, ceramides have been shown to possess an independent predictive value for negative (fatal, non-fatal, and overall) cardiovascular outcomes, including the onset of type 2 diabetes mellitus (T2DM) [7,8,9,10,11,12,13,14,15,16,17,18]. Based on the results of the abundant biomedical literature, an algorithm of cardiovascular disease (CVD) risk scoring was modeled by using three single ceramides and three ceramide/ceramide ratios, named CERT1 (ceramide test 1). In addition, with the aim to improve the performance of this score, another ceramide risk score, i.e., CERT2 (ceramide test 2), has been proposed, this being based on one ceramide/ceramide ratio, two ceramide/phosphatidylcholine (PC) ratios, and a single PC [6]. These ceramide risk scores (CERT1 and 2) have been tested in different clinical settings as a valuable (easy-to-use) tool for primary and secondary prevention of CVDs, including coronary artery disease (CAD), chronic heart failure (CHF), atrial fibrillation (AF), stroke, and T2DM [6].

A direct cause–effect relationship between CVDs and ceramides has not been fully established to date, though the pathophysiological involvement of ceramides in molecular processes of atherosclerosis may be prominent [19,20]. Furthermore, total cholesterol (T-C) and low-density lipoprotein cholesterol (LDL-C) seem to have a lower predictivity for CVD risk than ceramides (or ceramide ratios) [8].

As ceramide-specific medications are being developed [20,21], conventional strategies such as lipid-lowering pharmacotherapy and lifestyle interventions (e.g., body weight reduction programs, BWRP) can be administered to successfully reduce overall CVD risk [22,23]. Thus, ceramides and, particularly, CERT1 might identify patients at a high CVD risk in need of more intense medical supervision and pharmacological or non-pharmacological treatments [6].

Among the different categories of patients having a high CVD risk, CERT1 might be used as a decision-making tool in diagnostic stratification and primary prevention in essential obesity and, still more, metabolic syndrome, a topic that to our best knowledge has not been fully investigated so far, despite the well-known limitations associated with the use of the (standard) diagnostic criteria such as those defined by the International Diabetes Federation (IDF) [24]. Considering the need to improve the adherence exhibited by subjects with obesity administered with BWRPs [25], CERT1 might represent a motivational drive for an uncompliant patient.

The aim of the present study was to evaluate the diagnostic value of CERT1 in a group of subjects with obesity without (OB-MetS−) or with (OB-MetS+) metabolic syndrome, compared to an age- and sex-matched group of normal-weight (NW) subjects. Being a cross-sectional clinical study, Framingham risk score (FRS) and vascular age (VA) were used as (internationally validated) surrogates of CVD risk.

## 2. Results

Table 1 shows the demographic, biochemical, and clinical characteristics of the population recruited in the present study, subdivided into the three groups NW (n = 30), OB-MetS− (n = 24) and OB-MetS+ (n = 30), for a total of 84 subjects. Shortly, body mass index (BMI), waist circumference (WC), systolic blood pressure (SBP), diastolic blood pressure (DBP), insulin, homeostatic model assessment of insulin resistance (HOMA-IR), triglycerides (TG), and C reactive protein (CRP) were significantly higher in subjects with obesity compared to the NW ones (*p* < 0.05), with no differences between OB-MetS− and OB-MetS+ groups, with the exception of SBP, which was significantly higher in the latter than the former one (*p* < 0.05). High-density lipoprotein cholesterol (HDL-C) levels were significantly lower in OB-MetS− and OB-MetS+ groups in comparison with the NW one (*p* < 0.05).

When considering ceramides, plasma concentrations of Cer 18:0 were significantly higher in both OB-MetS− and OB-MetS+ subjects than NW ones (*p* < 0.05), with those of Cer 24:1 being significantly higher only in the OB-MetS+ group (*p* < 0.05). On the contrary, plasma concentrations of Cer 24:0 were significantly lower in both OB-MetS− and OB-MetS+ subjects than NW ones (*p* < 0.05) (Table 1).

After calculating ceramide ratios, Cer 16:0/24:0, Cer 18:0/24:0, and Cer 24:1/24:0 were significantly higher in obese subjects than in NW ones (*p* < 0.05), with no differences between OB-MetS− and OB-MetS+ groups (Table 1).

**Figure 1:**
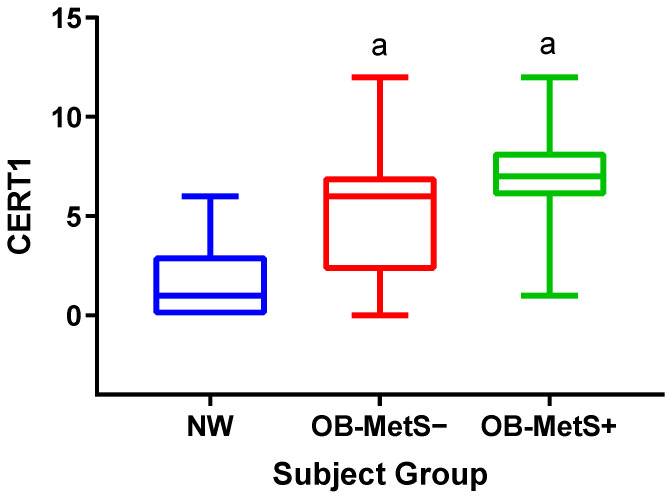
Ceramide risk score (CERT1) in the three groups: NW, OB-MetS−, and OB-MetS+. ^a^: *p* < 0.05 vs. NW. For abbreviations, see the text.

Figure 1 shows the median values of CERT1 in the three groups, with those in the OB-MetS− and OB-MetS+ groups being higher than in the NW one (*p* < 0.05). There was no statistical difference in CERT1 between OB-MetS− and OB-MetS+ subjects.

Table S1, included in the Supplementary Material, reports the results of a series of multiple linear regressions modeled by inserting plasma concentrations of ceramides (i.e., Cer 16:0, Cer 18:0, Cer 24:1, and Cer 24:0), ceramide ratios (i.e., Cer 16:0/24:0, Cer 18:0/24:0, and Cer 24:1/24:0) or CERT1 as the dependent variable and WC, SBP/DBP, HOMA-IR, HDL-C, TG, and CRP as independent variables. While only CRP significantly predicted Cer 16:0 (*p* < 0.05), significant predictors of both Cer 18:0 and Cer 24:1 were WC, SBP, and HOMA-IR (*p* < 0.05). In the model of multiple linear regression, only HDL-C significantly predicted Cer 24:0. There were significant associations of Cer 16:0/24:0, Cer 18:0/24:0, and Cer 24:1/24:0 with DBP (*p* < 0.05). Finally, CERT1 was significantly predicted by WC, HOMA-IR, and CRP (*p* < 0.05).

Table S2, included in the Supplementary Material, summarizes the results of a series of ANCOVAs, in which CERT1 was compared among the three groups after adjustment with WC, HOMA-IR, and/or CRP, the unique independent variables that were significantly associated as shown by the previous model of multiple linear regression. Shortly, the significant difference in CERT1 between the OB-MetS− or OB-MetS+ and NW groups, evidenced by ANOVA previously described (Figure 1), was maintained when the covariates HOMA-IR and CRP were simultaneously considered (i.e., HOMA-IR + CRP). The adjustments of CERT1 for WC + HOMA-IR or WC + CRP did yield a loss of the statistical significance of the difference in CERT1 between the OB-MetS+ and NW groups or between the OB-MetS− and NW groups. When CERT1 was adjusted for only WC, the result of the corresponding ANCOVA showed the maintenance of the significant difference between the OB-MetS+ and NW groups, but not between the OB-MetS− and NW groups.

**Figure 2:**
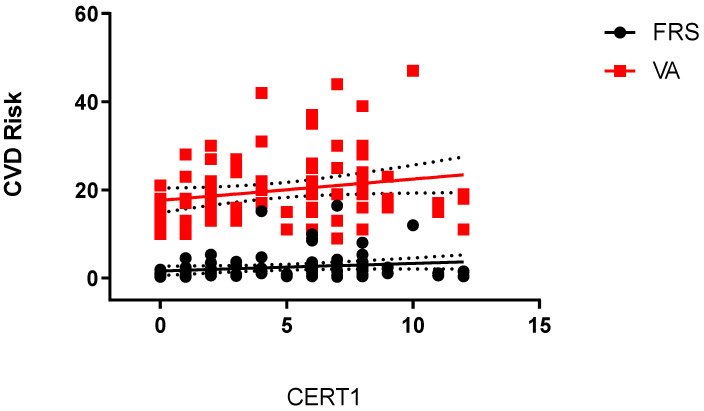
Linear regressions of FSR or VA with CERT1. For abbreviations, see the text.

Framingham risk score (FRS) and vascular age (VA) were significantly higher in OB-MetS+ subjects compared to NW or OB-MetS− ones (*p* < 0.05), with no significant differences in FRS and VA between the NW and OB-MetS− groups (Table 1). While there was no significant correlation between CERT1 and FRS (r = 0.1962; *p* = 0.0737), CERT1 was significantly correlated with the VA (r = 0.2173; *p* = 0.0470). The equations of the corresponding linear regressions were also calculated: (i) FRS = 0.169 × CERT1 + 1.635 (*p* = 0.0844); (ii) VA = 0.4843 × CERT1 + 17.61 (*p* = 0.0432) (Figure 2). By using these equations, the values of FRS and VA were calculated for each CERT1-based risk group (Tables S3 and S4 in the Supplementary Material).

**Figure 3:**
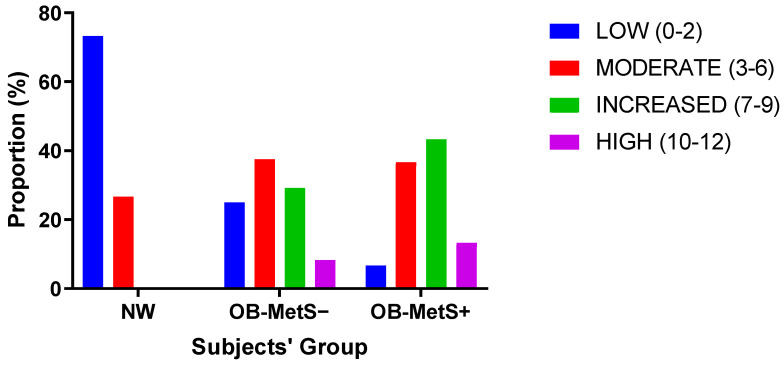
Proportions of NW, OB-MetS− and OB-MetS+ subjects, distributed according to the four CERT1-based risk groups (i.e., low, moderate, increased and high). For further information see also Tables S3 and S4 in the Supplementary Material. For abbreviations, see the text.

After having distributed the subjects of the study population according to the four CERT1-based risk groups (i.e., low, moderate, increased, and high risk; see Materials and Methods for further details), the proportions of the observations in the three groups (i.e., NW, OB-MetS−, and OB-MetS+) significantly varied from risk group to risk group (*chi*-square = 36.407 with 6 degrees of freedom, *p* < 0.001) (Figure 3).

## 3. Discussion

CERT1 represents an easy-to-use marker for primary and secondary prevention of CVDs, calculated by applying an algorithm based on plasma levels of specific ceramides (i.e., Cer 16:0, Cer 18:0, Cer 24:1, and Cer 24:0) and the values of the related ceramide ratios (i.e., Cer 16:0/24:0, Cer 18:0/24:0, and Cer 24:1/24:0), with quartiles as cut-offs and a categorial scoring [6].

In the present study, carried out in a population of OB-MetS− and OB-MetS+ subjects, compared to an age- and sex-matched NW group, CERT1 was higher in obese subjects than in NW ones. WC, HOMA-IR (surrogates of IDF diagnostic criteria for metabolic syndrome) [26] and CRP (a marker of inflammation) [27] were associated with CERT1, with the contribution of other parameters such as DBP/SBP, HDL-C and TG (i.e., arterial hypertension and dyslipidemia) being negligible.

Thus, CERT1 seems to connote the state of obesity independently from the diagnosis of metabolic syndrome. Furthermore, visceral adiposity, insulin-resistance, and chronic low-grade inflammation represent, among those herein considered, the main factors capable of explaining the increased CERT1 in both OB-MetS− and OB-MetS+ subjects.

Unfortunately, though higher, CERT1 values in OB-MetS+ subjects were not statistically different from those in OB-MetS− subjects. This result might be related to the reduced sample size of our study or, alternatively, to the intrinsic limitations of IDF diagnostic criteria that do not permit a full differentiation between those subjects at high CVD risk among OB-MetS− and OB-MetS+ groups [28]. For example, an OB-MetS− subject having an uncontrolled arterial hypertension might exhibit a higher CVD risk than an OB-MetS+ subject.

Due to the cross-sectional design of the present study, CVD risk was assessed by using two internationally validated methods of scoring, i.e., FRS and VA [29]. In particular, VA was associated with CERT1, with the association of FRS with CERT1 being near statistical significance. These results further confirm the validity of CERT1 as uniquely biochemical method for assessing CVD risk in an obese population independently from the diagnosis of metabolic syndrome [30].

Due to the involvement of ceramides in several molecular mechanisms underlying atherosclerosis, including the demonstration of high levels of ceramides in atherosclerotic plaques, and the ability of ceramides to promote endothelial uptake of LDL-C, superoxide anion production, inflammation, and apoptosis [19], CERT1 might be a valuable tool to better stratify subjects with obesity according to the individual CVD risk independently from the diagnosis of metabolic syndrome. In fact, in the present study, while no one in the NW group was categorized as an individual at increased or high risk, proportions of OB-MetS− and OB-MetS+ subjects were included in the categories at low and moderate risk, suggesting that CERT1, rather than the diagnosis of metabolic syndrome, might better identify the obese subjects that need more frequent medical supervision and, importantly, a more aggressive lifestyle change and pharmacological therapy, aimed at reducing the individual CVD risk [6].

Based on the results from the present study and the most recent biomedical literature on this topic [7,8,9,10,11,12,13,14,15,16,17,18], it is difficult to establish whether CERT1 may be an additional or substitutive marker to the currently used IDF diagnostic criteria for metabolic syndrome [26]. Nevertheless, the main advantage of CERT1 in comparison to the diagnosis of metabolic syndrome, which is simply dichotomous (OB-MetS− or OB-MetS+), relies on the continuity of the biochemical variables (i.e., ceramides and ceramide ratios), components of CERT1, with the possibility of having cut-off values (quartiles) and of monitoring patient’s clinical state before and after any intervention, simply requiring a blood test at the lab (i.e., measurement of plasma levels of ceramides).

Taking into account the results of ANCOVAs obtained in the present study, visceral adiposity (i.e., WC) appeared to be the *primum movens* of dysregulation of ceramide metabolism occurring during the pathophysiological *continuum* from morbid obesity (i.e., OB-MetS−) to metabolic syndrome (i.e., OB-MetS+), in which, in addition to visceral adiposity, chronic low-grade inflammation and insulin-resistance become dominant [31]. In fact, when adjusted uniquely for WC, the difference in CERT1 between the OB-MetS− and NW groups disappeared, while that between the OB-MetS+ and NW groups was maintained; moreover, when combining WC with CRP or HOMA-IR as covariates, i.e., visceral adiposity with chronic low-grade inflammation or insulin-resistance, it was possible to abate the difference in CERT1 between the OB-MetS+ and NW groups.

There is another reason to extend the use of CERT1 in clinical practice, particularly in the pharmacological management of the metabolic syndrome. In fact, several enzymes of sphingolipids synthesis have already been identified as potential drug targets [32]. For instance, myriocin, inhibitor of serine palmitoyl transferase (SPT); L-cycloserine and fumonisin B1, inhibitors of de novo ceramide synthesis by desaturase 1 (DES1); and L-alpha-phosphatidyl-D-myo-inositol-3,5-bisphosphate, inhibitor of acid sphingomyelinase (ASMase), represent compounds known to us to be effective in different experimental models of obesity, but, unfortunately, are still not available for clinical use [33,34]. Therefore, a CERT1-based stratification of the obese population might identify patients that will better benefit from these extremely promising drugs.

For obvious reasons, the present discussion has been focused on the clinical implications related to the use of CERT1, leaving a restricted room for the single ceramide species and the corresponding ratios to Cer 24:0. However, evidence is also accumulating on ceramide-chain-length-specific functions [35]. For instance, recently, it has been demonstrated that the relative increase in long-chain species (C18:0) but not in very-long-chain (C24:1) species are associated with insulin-resistance and apoptosis in animal models [36,37,38]. The results of the present study would confirm this view, as shown by the association of Cer 18:0 with HOMA-IR.

According to other studies [35], our results seem to suggest that long-chain species (e.g., Cer 16:0 and Cer 18:0) are more “harmful” than very-long-chain species, with, for example, Cer 24:0 being associated with no surrogate of IDF diagnostic criteria (i.e., WC, DBP/SBP, HOMA-IR, TG); on the contrary, in our model of linear regression, Cer 24:0 increased with plasma levels of HDL-C, which, as well-known, is cardioprotective [39]. Of note, some studies have shown that Cer 24:1 behaves differently from Cer 24:0, suggesting that additional molecular (dys)regulation in sphingolipid metabolism also takes place in pathophysiological conditions such as obesity and CVDs (e.g., alteration in the desaturation of acyl-groups) [8]. At the biochemical level, this altered composition of ceramide species might partially be explained by the existence of different isoforms of ceramide synthase (CerS), whose gene expressions change in pathophysiological conditions such as obesity and metabolic syndrome [35]. This would explain the differences between the single ceramides and the related ceramide ratios among NW, OB-MetS−, and OB-MetS+ subjects, recruited in the present study, further providing a biological rationale for the use of ceramide ratios and, then, CERT1 for the aims of the present study, focused on metabolic syndrome [40].

Finally, while the present study revealed an association between CERT1 and CVD risk (e.g., VA), particularly in metabolic syndrome, it will be a highly interesting topic for future investigations to establish whether ceramide composition within the diet of BWRPs (or uniquely weight loss) may result in a reduction of CERT1 and, consequently, CVD risk (or produce other non-CVD-related benefits) in subjects with obesity [6].

Some limitations of the present study should be mentioned:

(a) Being a cross-sectional study, CVD risk was calculated for each subject by means of FRS and VA, which mainly predict CVD risk related to CAD [29]; thus, the power of CERT1 in predicting CVD risk in metabolic syndrome is only presumptive; long-term, large-scale, and (importantly) prospective studies are mandatory for assessing the “real” hazard rate of CVD events in OB-MetS− and, particularly, OB-MetS+ subjects, categorized according to CERT1 groups;
(b) Since there is the intriguing view that ceramides may better stratify (only residual?) CVD risk than the cholesterol-related lipids such as LDL-C and HDL-C [8], we cannot rule out that different factors, rather than visceral adiposity (WC), chronic low-grade inflammation (CRP), and insulin-resistance (HOMA-IR), might affect CERT1, being pathophysiologically involved in the dysregulation of ceramide metabolism in obesity and metabolic syndrome;
(c) CERT2 has been proposed as an alternative to CERT1, with the former one being a more robust marker of CVD risk than the latter one [6]; unfortunately, in the present study, plasma levels of PCs, components of the algorithm used to calculate CERT2, were not measured; although we are willing to evaluate CERT2 in a future clinical study of ours, we believe that the new results will confirm the conclusions drawn by the use of only CERT1.

## 4. Materials and Methods

### 4.1. Subjects

Subjects with obesity (BMI > 35 kg/m^2^), hospitalized at the Division of Metabolic Diseases, Istituto Auxologico Italiano, Piancavallo-Verbania, Italy, for a 3-week multidisciplinary integrated BWRP, were recruited for the current study. NW (healthy) subjects, age- and sex-matched, selected among friends and relatives of the medical and nursing staff, were recruited as control group. Both obese and NW subjects were moderately active (60 min of physical activity, two times/week). All females were amenorrheic; the study was conducted in the menstrual cycle’s follicular phase.

After having verified exclusion criteria, particularly the existence of any disease apart from essential obesity, or assumption of any drug, clinical, biochemical, and anthropometric data were collected from each participant.

The study protocol was approved by the Ethical Committee (EC) of the Istituto Auxologico Italiano, IRCCS, Milan, Italy (EC code: 2021_02_23_11; research project: 01C126; acronym: SFINGOTRANSADIP); the protocol was explained to the subjects, who gave their written informed consent.

### 4.2. Anthropometric Measurements

A scale with a stadiometer was used to determine height and weight (Wunder Sa. Bi., WU150, Trezzo sull’Adda, Italy). WC was measured with a flexible tape in a standing position, halfway between the inferior margin of the ribs and the superior border of the crista. BMI was determined in all subjects.

### 4.3. Metabolic Parameters

Blood samples (about 10 mL) were collected at around 8:00 AM after an overnight fast (about 12h) at the beginning of the BWRP.

T-C, HDL-C, LDL-C, TG, glucose, insulin, and CRP were measured.

Calorimetric enzymatic assays (Roche Diagnostics, Monza, Italy) were used to determine serum T-C, LDL-C, HDL-C, and TG levels. The sensitivities of the assays were 3.86 mg/dL [1 mg/dL = 0.03 mmol/L], 3.87 mg/dL [1 mg/dL = 0.03 mmol/L], 3.09 mg/dL [1 mg/dL = 0.03 mmol/L], and 8.85 mg/dL [1 mg/dL = 0.01 mmol/L], respectively.

Serum glucose level was measured by the glucose oxidase enzymatic method (Roche Diagnostics, Monza, Italy). The sensitivity of the method was 2 mg/dL [1 mg/dL = 0.06 mmol/L].

Serum insulin concentration was determined by a chemiluminescent emu-no-metric assay, using a commercial kit (Elecsys Insulin, Roche Diagnostics, Monza, Italy). The sensitivity of the method was 0.2 µIU/mL [1 µU/mL = 7.18 pmol/L].

CRP was measured using an immunoturbidimetric assay (CRP RX, Roche Diagnostics GmbH, Mannheim, Germany). The sensitivity of the method was 0.03 mg/dL.

The intra- and inter-assay coefficients of variation (CVs) were the following: 1.1% and 1.6% for T-C, 1.2% and 2.5% for LDL-C, 1.8% and 2.2% for HDL-C, 1.1% and 2.0% for TG, 1.0% and 1.3% of glucose, and 1.5% and 4.9% of insulin.

For each patient, HOMA-IR was also calculated according to the following formula: (insulin [μIU/mL] × glucose [mmol/L])/22.5 [41].

### 4.4. Blood Pressure

Blood pressure was measured on the right arm, using a sphygmomanometer with appropriate cuff size, with the subject in a seated position and a relaxed condition. The procedure was repeated three times at 10 min intervals in-between; the means of the three values for SBP and DBP were recorded.

### 4.5. Definition of Metabolic Syndrome

According to the IDF criteria for diagnosis of metabolic syndrome in adults [26], subjects with obesity were considered positive for the presence of metabolic syndrome if they had three or more of the following factors: (i) abdominal obesity; (ii) hypertriglyceridemia or specific treatment for this lipid abnormality; (iii) reduced HDL-C levels or specific treatment for this lipid abnormality; (iv) arterial (systolic or diastolic) hypertension or treatment of previously diagnosed hypertension; (v) hyperglycemia or previously diagnosed T2DM.

### 4.6. Lipid Extraction and Ceramide Content Quantification

Sphingolipids extraction and targeted LC–MS/MS analysis were performed as previously described [42,43,44]. Sphingolipids were assayed in 25 µL of plasma, collected as above described. Plasma was diluted to a final volume of 100 µL with water, and after the addition of 850 µL methanol/chloroform mixture (2:1 *v*/*v*), samples were incubated for 1h at 38 °C. Then, to enhance their recovery, alkaline methanolysis was performed by incubation at 37 °C for 2 h with 75 µL of potassium hydroxide 1 M in methanol. After neutralization with 4 µL of pure acetic acid, samples were centrifuged (15 min at 13,400 RPM) and evaporated. The residues were dissolved in 100 µL of methanol, centrifuged for 10 min at 13,400 RPM, and withdrawn in a glass vial. Finally, samples were analyzed by LC Dionex 3000 UltiMate (Thermo Fisher Scientific, Waltham, MA, USA) coupled to a tandem mass spectrometer AB Sciex 3200 QTRAP (AB Sciex, Framingham, MA, USA). The separation was achieved by reversed-phase chromatography, using (specifically for ceramics) BEH C8 1.7 μm, 100 × 2.1 mm (Waters, Milford, MA, USA) mixing eluent A (0.2% formic acid 2 mM ammonium format water-solution) and eluent B (methanol, 0.2% formic acid 1 mM ammonium formate). Quantitative analysis was performed interpolating each peak area of analyte/area IS with a calibration curve for each sphingolipid.

### 4.7. Ceramide Risk Score

The ceramide risk score (CERT1) was calculated as reported by Hilvo et al. [6]. In brief, for each individual, plasma concentrations of specific ceramides (i.e., Cer 16:0, Cer 18:0, Cer24:0, and Cer 24:1) and the related ratios (i.e., Cer 16:0/24:0, Cer 18:0/24:0, and Cer 24:1/24:0) were determined at basal conditions. If the variable belonged to the 3rd quartile, the individual received +1 points, and, if to the 4th quartile, +2 points. The sum of the six subscores corresponded to the CERT1. Based on this parameter, the subjects were split into four risk categories (low = 0–2, moderate = 3–6, increased = 7–9, and high = 10–12). See also Tables S3 and S4, included in the Supplementary Material.

### 4.8. Calculation of Framingham Risk Score and Vascular Age

The 2008 Framingham risk score (FRS) assessment was employed to determine the CVD risk and, additionally, the FRS-based vascular age (VA) [29].

The FRS algorithm considers age, T-C, HDL-C, SBP, ongoing treatment of hypertension, smoking, and diabetes status, and provides sex-specific results.

VA is defined as a person’s age with the same predicted CVD risk, but with all other risk factor levels in the normal ranges.

### 4.9. Statistics

Sigma Stat 4.0 (SysStat Software Inc., Palo Alto, CA, USA), IBM SPSS Statistics 28.0.1.1. (SPS, Milan, Italy), and GraphPad PRISM 7.0a (La Jolla, CA, USA) were used for analyses and plotting.

Parameters were expressed as median [interquartile range] and analyzed by Kruskal–Wallis’s one-way analysis of variance (ANOVA) test, followed by the post-hoc Dunn’s test for multiple comparisons (NW vs. OB-MetS+ vs. OB-MetS+). Categorical variables were compared through *chi*-square or Fisher tests.

Multiple linear regression analysis was performed to investigate the associations between ceramides levels, ceramide ratios or CERT1 and some continuous clinical or biochemical variables (i.e., WC, SBP, DBP, HOMA-IR, TG, and HDL-C, which represent surrogates of IDF diagnostic criteria of metabolic syndrome [26], and CRP, which is a gross index of low-grade chronic inflammation in obesity [27]).

A non-parametric analysis of covariance (ANCOVA) was used to evaluate the contribution of WC, HOMA-IR, and/or CRP as covert(s) on the differences in CERT1 among NW, OB-MetS−, and OB-MetS+ subjects.

Spearman’s rank correlation coefficient was calculated to establish the relationship between CERT1 and FRS or VA; moreover, the equations of the corresponding linear regressions were deduced.

A *p*-value <  0.05 was considered statistically significant.

## 5. Conclusions

Based on the results of the present study carried out in an obese population with or without metabolic syndrome, compared with a sex- and age-matched NW subjects, we can conclude that:

(1) CERT1 is higher in obese than NW subjects, with no difference between the OB-MetS− and OB-MetS+ groups;
(2) WC, HOMA-IR, and CRP are predictors of CERT1, with the contribution of the other IDF criteria such as arterial hypertension and dyslipidemia being negligible;
(3) Adjustment for WC resulted in a loss of the difference in CERT1 between OB-MetS− and NW subjects, with the combination of WC and HOMA-IR or CRP as covariates being necessary to yield the same effect for the difference in CERT1 between OB-MetS+ and NW subjects;
(4) CERT1 is associated with VA;
(5) The proportions of NW, OB-MetS−, and OB-MetS+ subjects appeared to be distributed according to the CERT1-based risk groups (i.e., low, moderate, increased, and high risk).

Thus, based on the concept of CERT1, which is calculated by measuring specific ceramides and ceramide/ceramide ratios in the plasma, the clinical diagnosis of metabolic syndrome seems to be somewhat inaccurate in assessing CVD risk in an obese population. In fact, some OB-MetS− subjects would be at increased-high risk, while some OB-MetS+ subjects at low-moderate risk. 

Derangement of ceramide metabolism might depend on (molecular) pathophysiological mechanisms occurring in obesity that, differently from (macroscopic) CVD risk factors included in the clinical definition of metabolic syndrome, underlay the propensity and then the evolution towards CVDs. 

Due to the limitations of the present study, further prospective studies are needed before considering CERT1 as an additional or substitutive marker in clinical practice.

## Figures and Tables

**Table 1:** Demographic, clinical, and biochemical characteristics of the study population, subdivided into three groups: normal-weight (NW) and obese subjects without (OB-MetS−) or with (OB-MetS+) metabolic syndrome.

Parameter	NW	OB-MetS−	OB-MetS+
N.	(n = 30)	(n = 24)	(n = 30)
Sex (F/M)	19F-11M	18F-6M	19F-11M
Age (years)	29.15 [26.46; 33.14]	27.38 [21.35; 35.65]	30.43 [23.98; 41.18]
Smoking (yes/no)	7/23	9/15	9/21
BMI (kg/m^2^)	22.85 [20.79; 24.70]	42.88 [40.75; 119.25] ^a^	43.44 [41.53; 46.54] ^a^
WC (cm)	78.0 [76.3; 82.8]	110.0 [106.0; 119.3] ^a^	120.0 [113.3; 126.5] ^a^
SBP (mmHg)	120 [110; 120]	120 [120; 130] ^a^	130 [130; 140] ^a,b^
DBP (mmHg)	70 [70; 75]	80 [77.50; 80] ^a^	80 [80; 90] ^a^
Glucose (mg/dL)	87 [82.25; 94.25]	83 [80; 88.25]	86 [82.25; 94.75]
Insulin (mU/L)	6.65 [5.13; 8.80]	15.85 [11; 23.55] ^a^	25.05 [19.08; 30.25] ^a^
HOMA-IR	1.54 [1.07; 1.84]	3.23 [2.23; 4.68] ^a^	5.30 [4.32; 6.21] ^a^
T-C (mg/dL)	173 [158; 200.50]	160.50 [133.25; 188.50]	163 [148; 196]
HDL-C (mg/dL)	65 [56.25; 70.75]	45.50 [39.50; 50.25] ^a^	37.50 [32.50; 43.75] ^a^
LDL-C (mg/dL)	106.50 [86.25; 120.50]	101.50 [77.75; 122.25]	107.50 [96; 124.75]
TG (mg/dL)	63 [53; 85.75]	96 [85.75; 123.25] ^a^	125.50 [103.50; 159.25] ^a^
CRP (mg/dL)	0.10 [0; 0.20]	0.50 [0.28; 1.03] ^a^	0.55 [0.40; 1.08] ^a^
Ceramides (µmol/L)			
Cer 16:0	0.4463 [0.3834; 0.5280]	0.4359 [0.4025; 0.4727]	0.4533 [0.3815; 0.5484]
Cer 18:0	0.0663 [0.0557; 0.0790]	0.1121 [0.0794; 0.1377] ^a^	0.1242 [0.1053; 0.1714] ^a^
Cer 24:1	0.7663 [0.5601; 0.9362]	0.9581 [0.8408; 1.1890]	1.1021 [0.8999; 1.3634] ^a^
Cer 24:0	3.7086 [2.9225; 4.0040]	2.1955 [1.7563; 3.1066] ^a^	2.3265 [1.9734; 3.0387] ^a^
Ceramide Ratios			
Cer 16:0/24:0	0.1296 [0.1116; 0.1438]	0.1896 [0.1520; 0.2371] ^a^	0.1859 [0.1706; 0.2285] ^a^
Cer 18:0/24:0	0.0187 [0.0158; 0.0241]	0.0454 [0.0355; 0.0655] ^a^	0.0557 [0.0480; 0.0654] ^a^
Cer 24:1/24:0	0.2190 [0.1928; 0.2375]	0.4044 [0.3280; 0.5424] ^a^	0.4660 [0.4076; 0.5187] ^a^
CVD Risk			
FRS (%)	1.000 [0.675; 1.350]	1.150 [0.600; 1.675]	2.450 [1.100; 4.325] ^a,b^
VA (years)	16.000 [13.750; 18.000]	16.000 [13.250; 19.750]	23.500 [17.750; 30.000] ^a,b^

Note: Data, expressed as median and interquartile range [25th and 75th], were analyzed by Kruskal–Wallis’s one-way ANOVA test, followed by the post-hoc Dunn’s test for multiple comparisons. ^a^: <0.05 vs. NW group; ^b^: <0.05 vs. OB-MetS−. For abbreviations, see the text.

## Data Availability

The datasets used and/or analyzed in the present study are available from the corresponding author upon a reasonable request. Raw data will be uploaded to www.zenodo.org (accessed on 1 August 2023). immediately after the acceptance of the manuscript.

## Supplementary Materials

The support information can be downloaded at: https://www.mdpi.com/article/10.3390/ijms241512452/s1. Reference [45] is cited in Supplementary Materials.

## Abbreviations

AF, atrial fibrillation; ANCOVA, analysis of covariance; ANOVA, analysis of variance; ASMase, acid sphingomyelinase; BMI, body mass index; BWRP, body weight reduction program; CAD, coronary artery disease; Cer, ceramide; CerS, ceramide synthase; CERT1 or 2, ceramide test 1 or 2; CHF, chronic heart failure; CRP, C-reactive protein; CVD, cardiovascular disease; DBP, diastolic blood pressure; DFE, degrees of freedom for error; DES1, desaturase 1; DFH, degrees of freedom for the hypothesis, EC, Ethical Committee; FRS, Framingham risk score; HDL-C, high-density lipoprotein cholesterol; HOMA-IR, homeostasis model assessment of insulin-resistance; IDF, International Diabetes Federation; LC-MS/MS, liquid chromatography–tandem mass spectrometry; LDL-C, low-density lipoprotein cholesterol; NW, normal-weight; OB-MetS−, obese subjects without metabolic syndrome; OB-MetS+, obese subjects with metabolic syndrome; PC, phosphatidylcholine, SBP, systolic blood pressure; std, standard; SPT, serine palmitoyl transferase; T2DM, type 2 diabetes mellitus; T-C, total cholesterol; TG, triglycerides; VA, vascular age; VIF, variance inflation factor; WC, waist circumference.
