# Supplementary material for: Ceramide Risk Score in the Evaluation of Metabolic Syndrome: An Additional or Substitutive Biochemical Marker in the Clinical Practice?
Source: Int J Mol Sci. 2023 Aug 5;24(15):12452. doi: 10.3390/ijms241512452 (PMC10420317; doi:10.3390/ijms241512452)
Supplement: Supplementary file 1 [file ijms-24-12452-s001.zip › Table S1.pdf]

Table S1. Multiple linear regression with ceramides, ceramide ratios or ceramide risk score (CERT1) as dependent variable and WC, SBP/DBP, HOMA-IR, HDL-C and TG (surrogates of IDF diagnostic criteria of metabolic syndrome), and CRP (marker of inflammation) as independent variables.

Cer 16:0

	Coefficient	*Std. Error*	*t*	*P*	*VIF*
Constant	0.537	0.173	3.109	0.003	
WC	-0.000968	0.00107	-0.907	0.367	3.476
SBP	-0.00186	0.00151	-1.237	0.220	2.478
DBP	0.00219	0.00195	1.123	0.265	2.291
HOMA-IR	0.00723	0.00646	1.119	0.267	1.864
HDL-C	0.000364	0.00119	0.306	0.760	2.583
TG	0.000136	0.000285	0.478	0.634	1.567
CRP	0.0484	0.0237	2.039	0.045	1.380

Cer 18:0

	Coefficient	*Std. Error*	*t*	*P*	*VIF*
Constant	0.0901	0.0664	1.356	0.179	
WC	0.00101	0.000410	2.459	0.016	3.476
SBP	-0.00130	0.000579	-2.250	0.027	2.478
DBP	0.000601	0.000750	0.801	0.426	2.291
HOMA-IR	0.00638	0.00248	2.571	0.012	1.864
HDL-C	-0.0000399	0.000457	-0.0872	0.931	2.583
TG	-0.00000781	0.000109	-0.0714	0.943	1.567
CRP	0.0154	0.00913	1.683	0.096	1.380

Cer 24:1

	Coefficient	*Std. Error*	*t*	*P*	*VIF*
Constant	0.562	0.460	1.222	0.226	
WC	0.00786	0.00284	2.765	0.007	3.476
SBP	-0.00956	0.00401	-2.384	0.020	2.478
DBP	0.00404	0.00519	0.777	0.439	2.291
HOMA-IR	0.0378	0.0172	2.201	0.031	1.864
HDL-C	0.00403	0.00316	1.274	0.207	2.583
TG	0.000816	0.000757	1.077	0.285	1.567
CRP	0.0654	0.0632	1.034	0.304	1.380

Cer 24:0

	Coefficient	*Std. Error*	*t*	*P*	*VIF*
Constant	3.022	1.523	1.984	0.051	
WC	-0.00492	0.00941	-0.523	0.603	3.476
SBP	-0.0213	0.0133	-1.603	0.113	2.478
DBP	0.0183	0.0172	1.065	0.290	2.291
HOMA-IR	0.00201	0.0569	0.0352	0.972	1.864
HDL-C	0.0258	0.0105	2.458	0.016	2.583
TG	0.00298	0.00251	1.190	0.238	1.567
CRP	-0.0728	0.209	-0.348	0.729	1.380

Cer 16:0/24:0

	Coefficient	*Std. Error*	*t*	*P*	*VIF*
Constant	0.777	0.446	1.743	0.085	
WC	0.000399	0.00276	0.145	0.885	3.476
SBP	0.00467	0.00389	1.202	0.233	2.478

DBP	-0.0138	0.00504	-2.744	0.008	2.291
HOMA-IR	0.00796	0.0167	0.478	0.634	1.864
HDL-C	-0.00341	0.00307	-1.113	0.269	2.583
TG	-0.0000362	0.000734	-0.0493	0.961	1.567
CRP	0.0545	0.0613	0.889	0.377	1.380

**Cer 18:0/24:0**					
	Coefficient	*Std. Error*	*t*	*P*	*VIF*
Constant	0.0936	0.0484	1.932	0.057	
WC	0.000433	0.000299	1.447	0.152	3.476
SBP	0.000274	0.000422	0.648	0.519	2.478
DBP	-0.00137	0.000547	-2.503	0.014	2.291
HOMA-IR	0.00316	0.00181	1.745	0.085	1.864
HDL-C	-0.000617	0.000333	-1.852	0.068	2.583
TG	-0.0000495	0.0000797	-0.621	0.537	1.567
CRP	0.00703	0.00665	1.057	0.294	1.380

**Cer 24:1/24:0**					
	Coefficient	*Std. Error*	*t*	*P*	*VIF*
Constant	0.605	0.335	1.805	0.075	
WC	0.00370	0.00207	1.786	0.078	3.476
SBP	0.00198	0.00292	0.677	0.500	2.478
DBP	-0.0101	0.00379	-2.661	0.009	2.291
HOMA-IR	0.0195	0.0125	1.553	0.124	1.864
HDL-C	-0.00310	0.00231	-1.344	0.183	2.583
TG	-0.0000161	0.000552	-0.0291	0.977	1.567
CRP	0.0432	0.0461	0.938	0.351	1.380

**CERT1**					
	Coefficient	*Std. Error*	*t*	*P*	*VIF*
Constant	1.994	3.748	0.532	0.596	
WC	0.0620	0.0232	2.678	0.009	3.476
SBP	-0.0325	0.0327	-0.996	0.323	2.478
DBP	-0.00162	0.0423	-0.0383	0.970	2.291
HOMA-IR	0.439	0.140	3.134	0.002	1.864
HDL-C	-0.0324	0.0258	-1.257	0.213	2.583
TG	-0.00396	0.00617	-0.641	0.523	1.567
CRP	1.093	0.515	2.123	0.037	1.380

Note: for abbreviations see the text.
