# Supplementary material for: Ceramide Risk Score in the Evaluation of Metabolic Syndrome: An Additional or Substitutive Biochemical Marker in the Clinical Practice?
Source: Int J Mol Sci. 2023 Aug 5;24(15):12452. doi: 10.3390/ijms241512452 (PMC10420317; doi:10.3390/ijms241512452)
Supplement: Supplementary file 1 [file ijms-24-12452-s001.zip › Table S2.pdf]

Table S2. ANOCVA of ceramide risk score (CERT1) with WC. HOMA-IR and/or CRP as covariate/s.

**ANCOVA: adjustment for WC**			
F	*DFH*	*DFE*	*P Value*
2.312	2	81	0.106
Comparison	t	DF	P Value
NW vs. OB-MetS-	-1.234	81	0.221
NW vs. OB-MetS+	-2.135	81	0.036
OB-MetS- vs. OB-MetS+	-0.779	81	0.438

**ANCOVA: adjustment for HOMA-IR**			
F	*DFH*	*DFE*	*P Value*
5.543	2	81	0.006
Comparison	*t*	*DF*	*P Value*
NW vs. OB-MetS-	-2.616	81	0.011
NW vs. OB-MetS+	-3.050	81	0.003
OB-MetS- vs. OB-MetS+	-0.260	81	0.795

**ANCOVA: adjustment for CRP**			
F	*DFH*	*DFE*	*P Value*
9.229	2	81	0.000
Comparison	t	DF	P Value
NW vs. OB-MetS-	-3.130	81	0.002
NW vs. OB-MetS+	-4.071	81	0.000
OB-MetS- vs. OB-MetS+	-0.708	81	0.481

**ANCOVA: adjustment for WC + CRP**			
F	*DFH*	*DFE*	*P Value*
1.326	2	81	0.271
Comparison	*t*	*DF*	*P Value*
NW vs. OB-MetS-	-1.160	81	0.249
NW vs. OB-MetS+	-1.555	81	0.124
OB-MetS- vs. OB-MetS+	-0.306	81	0.760

**ANCOVA: adjustment for WC + HOMA-IR**			
F	*DFH*	*DFE*	*P Value*
0.785	2	81	0.459
Comparison	*t*	*DF*	*P Value*
NW vs. OB-MetS-	-1.041	81	0.301
NW vs. OB-MetS+	-1.106	81	0.272
OB-MetS- vs. OB-MetS+	-0.002	81	0.999

**ANCOVA: adjustment for CRP + HOMA-IR**			
F	*DFH*	*DFE*	*P Value*
4.009	2	81	0.022
Comparison	*t*	*DF*	*P Value*
NW vs. OB-MetS-	-2.328	81	0.022
NW vs. OB-MetS+	-2.520	81	0.014
OB-MetS- vs. OB-MetS+	-0.048	81	0.962

Note: for abbreviations see the text.
