# Supplementary material for: Ceramide Risk Score in the Evaluation of Metabolic Syndrome: An Additional or Substitutive Biochemical Marker in the Clinical Practice?
Source: Int J Mol Sci. 2023 Aug 5;24(15):12452. doi: 10.3390/ijms241512452 (PMC10420317; doi:10.3390/ijms241512452)
Supplement: Supplementary file 1 [file ijms-24-12452-s001.zip › Tables S3-S4.pdf]

Tab. S3. SCORE CALCULATION: Ceramide Risk Score (CERT1)

Score Component	Q1	Q2	Q3	Q4
Cer 16:0	0	0	+1	+2
Cer 18:0	0	0	+1	+2
Cer 24:1	0	0	+1	+2
Cer 16:0/24:0	0	0	+1	+2
Cer 18:0/24:0	0	0	+1	+2
Cer 24:1/24:0	0	0	+1	+2
**CERT1 = sum of the six "score components"**				

Tab. S4. RISK GROUP DETERMINATION: Ceramide Risk Score (CERT1), Framingham Risk Score (FRS) and Vascular Age (VA)

Risk Group	CERT1	FRS (%)	VA (years)
Low	0-2	1.64-3,02	17.6-18.6
Moderate	3-6	3.71-5.78	19.1-20.5
Increased	7-9	6.47-7.85	21.0-22.0
High	10-12	8.54-9.92	22.5-23.4

Tabs. S1-S2. Calculation of ceramide risk score (CERT1) and determination of CVD risk groups (*). When determining the score for a subject, it is evaluated to which quartile the person belongs with regards to each score component. In particular, CERT1 has six components, and only quartiles 3 and 4 give points. Thus, the scale for this score ranges from 0 to 12 points. Based on this, the subject is categorized to one of the CVD risk categories. Please, note that: (1) the quartiles have been calculated by using all data of the present study; (2) FRS and VA represent the expected values, calculated by using the equations of the corresponding linear regressions of FRS or VA with CERT1. See the text for further details.

*Hilvo, M.; Meikle, P.J.; Pedersen, E.R.; Tell, G.S.; Dhar, I.; Brenner, H.; Schöttker, B.; Lääperi, M.; Kauhanen, D.; Koistinen, K.M.; et al. Development and validation of a ceramide- and phospholipid-based cardiovascular risk estimation score for coronary artery disease patients. *Eur. Heart J.* **2020**, *41*, 371–380. doi: 10.1093/eurheartj/ehz387.
